# Draft Genome Sequence of *Enterococcus hirae* Strain INF E1 Isolated from Cultured Milk

**DOI:** 10.1128/genomeA.00498-14

**Published:** 2014-07-17

**Authors:** Davide Porcellato, Hilde M. Østlie, Siv B. Skeie

**Affiliations:** Department of Chemistry, Biotechnology and Food Science, Norwegian University of Life Sciences, Aas, Norway

## Abstract

Here, we present the draft genome of *Enterococcus hirae* INF E1, found as a contaminant in cultured milk and studied for its ability to metabolize milk fat globule membrane glycoconjugates.

## GENOME ANNOUNCEMENT

The finding of the genus *Enterococcus* in the cheese environment is controversial. Its presence is considered essential for flavor development in some Mediterranean cheese varieties, while in most other cheeses it is considered negative, as some strains of this genus are opportunistic pathogens (1, 2). The main source of *Enterococcus* spp. in dairy products is the environmental contamination of milk, and in case of traditional fermented cheese made from raw milk the presence of *Enterococcus* spp. has been well described (3, 4). Some *Enterococcus* strains isolated from the dairy environment have previously shown good adaptability to grow in cheese environments as nonstarter lactic acid bacteria (5). Metabolic studies have shown interesting features of a cultured milk isolate of *Enterococcus hirae* (INF E1) regarding its ability to utilize most milk fat globule membrane monosaccharides (6) and to survive the harsh conditions in the gastrointestinal tract (7). This suggests that *E. hirae* INF E1 survives in high numbers during cheese ripening and enters the intestine in good shape.

Here, we present the draft genome sequence of the cultured milk isolate *E. hirae* INF E1. Good-quality DNA was submitted for sequencing on an Illumina MiSeq platform at the Norwegian Sequencing Center (University of Oslo, Oslo, Norway) with 150-fold coverage of the genome. High-quality reads were *de novo* assembled using CLC Genomics Workbench 5.5 (CLC bio). Contigs obtained from the assembler and with lengths over 1,000 bp and coverages over 20× were oriented and ordered against the complete genome sequence of *E. hirae* ATCC 9790 using progressiveMauve (8). Coding DNA sequences (CDS) were predicted and annotated using the NCBI Prokaryotic Genome Annotation Pipeline (9).

The draft genome of *E. hirae* INF E1 consists of 22 contigs, for a total of 2,807,725 bp with a GC content of 36.8%. The largest contig is 560,880 bp. The total number of coding sequences (CDS) was 2,404 and the number of RNAs was 53. A plasmid of length 5,376 bp was found and encoded 7 hypothetical proteins. Two confirmed clustered regularly interspaced short palindromic repeat (CRISPR) arrays were identified using CRISPRfinder (10).

Interestingly, three gene clusters for the production of secondary metabolites were identified using the Antibiotics and Secondary Metabolite Analysis Shell (11). The three gene clusters encode proteins for the production of a putative class II lantipeptide, a terpene, and a bacteriocin. The construction of the metabolic model for *E. hirae* INF E1 will help to elucidate the possible energy sources and metabolic pathways present in this isolate and the biological importance and adaptation of this species in the dairy environment.

### Nucleotide sequence accession numbers.

This whole-genome shotgun project has been deposited at DDBJ/EMBL/GenBank under the accession number JMIG00000000. The version described in this paper is version JMIG01000000.
